# Reconciling the ideals of open science with data privacy in the context of health research in Nigeria: A legal analysis

**DOI:** 10.21203/rs.3.rs-3293485/v1

**Published:** 2023-10-24

**Authors:** Aishatu Eleojo Adaji

**Affiliations:** Osun State University College of Law

**Keywords:** consent, data protection, health research, open science, personal information, privacy

## Abstract

**Background:**

The Nigerian Revised National Policy on Science, Technology and Innovation 2022 formally proposed the adoption of open science principles among researchers and all other stakeholders in Nigeria. With regard to health research, open science would allow the dissemination and sharing of scientific data and other research outputs among health researchers, thereby accelerating the scientific process and the development of innovative solutions for health challenges. However, in this context, the existing privacy and data protection rules can profoundly affect the adoption and sustainability of open science in Nigeria. This is because researchers rely heavily on accessing and sharing personal information and the biological samples of human subjects. Therefore, this study identifies and examines possible legal issues and challenges arising from the existing privacy and data protection rules as researchers adopt an open science approach to health research in Nigeria.

**Methods:**

This study adopts a qualitative approach, providing a legal analysis of existing national, regional and international instruments on privacy, data protection and health research in the context of open science in Nigeria. The study also draws on relevant research and non-research articles on open science, health research, privacy and data protection published in English. Searches for the articles were conducted on various sites through the Google search engine, using terms such as ‘sharing of data’, ‘data privacy’, ‘principles of data protection’, ‘open science’, etc.

**Results/Conclusion:**

The study finds that the right to privacy and data protection could affect the promotion, adoption and sustainability of open science among health researchers in Nigeria, as well as their ability to engage in open collaborative research with their peers in Africa and other jurisdictions. To prevent infringements of the privacy and data protection rules by researchers and thereby ensure the adoption of open science approaches in health research, the study further identifies various legal options for researchers, including using personal data or material transfer arrangements, which, among other things, require recipients or users of human data or biological samples to commit not to re-identify human subjects.

## Background

As the world grapples with existing and emerging health challenges, the demand for access to scientific knowledge, data, and other intellectual resources and collaborative scientific research, innovation and technological development is increasing. The recent coronavirus disease (Covid-19) pandemic further heightened this demand. In this regard, open science has been increasingly recognized as a global tool for driving science, technology and innovation [1]. This is because open science is characterized by greater access to and sharing of research tools, data and other valuable research outputs and broader collaboration in the research, innovation and development processes. Thus, it is believed that it can speed up access to new and innovative technological solutions needed to address complex and unending global challenges such as climate change, diseases and food insecurity, as well as to reduce the expanding technological and knowledge gaps between countries [1]. With specific regard to health, its potential to facilitate equitable access to vaccines and other health technology is well acknowledged [2, 3].

To facilitate the adoption of open science in Nigeria, a set of strategies was formulated in the recently Revised National Policy on Science, Technology and Innovation, launched in March 2022 [4, 10]. This latest National Science, Technology and Innovation Policy (NSTIP 2022) [10] is the first significant policy framework to support and promote open science in Nigeria. It is influenced by the UNESCO Recommendation on Open Science 2021, an international framework promoting open science policy and practice. While NSTIP 2022 suggests a commitment to the advancement of open science in Nigeria, a number of legal issues may need to be addressed to ensure its implementation and sustainability, especially in the context of health research. This is mainly because open science involves the use, sharing and collaborative development of a wide range of tangible and intellectual resources, such as scientific information and data, publications, research tools and physical samples – some of which may be subject to third-party rights or other forms of restrictions under various existing legal frameworks in Nigeria.

More specifically, the collection, use or reuse and sharing of biological samples and human data, including genetic data and information about human health, by researchers who subscribe to open science raise several issues concerning privacy and data protection under existing policies and regulatory frameworks in Nigeria. These include whether, in adopting open science in health research, researchers can *publicly* or *openly* disclose and share their research data or outputs and if such action can be reconciled with existing legal regimes on privacy rights and data protection in Nigeria.

Although the NSTIP 2022 seeks to promote open science, it does not go far enough to address the foregoing legal issues. Therefore, to address the gap, this study examines how the right to privacy and data protection could affect the promotion, adoption and sustainability of open science among health researchers in Nigeria. The study addresses the following questions: To what extent could data privacy rights impede open science among health researchers in Nigeria? And how can ‘open sharing’ be sustained in health research without undermining the privacy and other related rights of the human subjects guaranteed under existing legal regimes in Nigeria?

### Open science trends and opportunities

Although the concept of open science is not new, it has rapidly gained traction since the Covid-19 pandemic [2, 3]. Historically, scientific research practices, particularly during the European scientific revolution of the 16th and 17th centuries, were driven mainly by the idea that scientific knowledge is community property or public good [3]. In this context, the timely communication of scientific findings and the exchange of ideas among scientists at conferences organized by scientific societies and through publication in journals were considered significant parts of the scientific research process. They enabled researchers to reproduce or challenge scientific claims and engage in follow-up research, thereby fostering the credibility and advancement of science [5].

Over time, several initiatives supporting the sharing of and unrestricted access to software codes, scientific literature, research data, and other intellectual and tangible resources emerged to speed up innovation and increase the societal impact of science. Various concepts have been used to capture these initiatives, including *open source, open access* and *open data*, among others [1]. Underpinning these initiatives is the shared belief that openness and transparency bring about maximum utilization of research outputs and provide the foundations upon which others can build, among other things. Facilitated by these initiatives, there is also a push for a collaborative approach to the scientific research process. This is because it is believed that science has become interdisciplinary in nature and that global challenges require collaborations among researchers and stakeholders to expedite the scientific research, innovation and development process [1].

The term *open science* is increasingly being used to reflect the convergence of the diverse open and *collaborative* practices in science. In other words, open science means more than just the sharing of software and source codes (as in the open source movement) or publications (as in the open access movement) or research data (as in the open data movement). Rather it is all of these and more, with open science opening the research process to allow collaboration beyond the traditional scientific community. The earlier movements reflect the different aspects of modern open science and, as such, are now better understood as its pillars [2, 6]. In this vein, various international [1], regional [7], and national standards [8, 9] are emerging to define and facilitate the open science movement. While some are broad, as in the case of the UNESCO Recommendation on Open Science 2021, others may be limited in terms of the scope of application, applying only to a particular sector such as the public sector, or dealing with an aspect of open science like open access to publications.

### The UNESCO Recommendation on Open Science 2021 defines open science as:

an inclusive construct that combines various movements and practices aiming to make multilingual scientific knowledge openly available, accessible and reusable for everyone, to increase scientific collaborations and sharing of information for the benefits of science and society, and to open the processes of scientific knowledge creation, evaluation and communication to societal actors beyond the traditional scientific community. It comprises all scientific disciplines and aspects of scholarly practices, including basic and applied sciences, natural and social sciences and the humanities .… [1]

In the context of the UNESCO Recommendation on Open Science 2021, it is understood that, while the public sector plays a leading role in implementing open science, privately funded research could also be subject to the guiding principles of open science.^1^

In Nigeria, the NSTIP 2022 [10] merely acknowledged that open science allows scientific information, data and outputs to be widely accessible and more reliably harnessed with the active engagement of all stakeholders.^2^ Through this Policy, the Nigerian government proposes to make publicly funded research outputs and resources available to all while ‘encouraging Nigerians’, in general, to engage in open science. It is unclear whether or how the policy will apply to the private sector. Even so, the potential to facilitate scientific collaboration among researchers and to accelerate health research and innovation in Nigeria through open science cannot be underestimated. This is of significant import as diseases primarily affecting Nigeria and other African countries remain under-researched [11].

The fundamental principles of transparency, equality, accountability, collaboration, flexibility and sustainability are integral to the effective implementation of modern open science [1]. These principles are founded on the core values of quality and integrity, collective benefit, equity and fairness, as well as diversity and inclusiveness [1]. Thus, this study examines how relevant regulatory frameworks on privacy rights and data protection in Nigeria implicate these values and principles vis-a-vis open science in health research.

## Methods

This study is qualitative research which consisted of a legal analysis of how the existing frameworks on privacy and data protection at the national and international levels implicate health research in the open science context. The search for relevant legal frameworks was carried out largely on the official websites of the Nigerian government, including that of the Nigeria Data Protection Commission, the European Union (EU) and the United Nations (UN), among others, to ensure the accuracy of the information obtained. Other secondary sources of information in English, such as journal articles, newspaper articles and reports, were also considered. For this purpose, Google searches were conducted using a broad range of terms, including ‘sharing of data’ or ‘data sharing’, ‘consent’, ‘data protection’, ‘health research’, ‘open science’, ‘open data’, ‘open access’, ‘anonymized data’, ‘synthetic data’, etc.

## Discussion

Scientific research is increasingly data-driven. Regarding the open science movement, open data is considered an integral part of the health research process. Bearing in mind the core values and principles of open science, such as inclusiveness, openness in this context means more than making diverse data accessible, reusable, combinable and transferrable. It also means that the process through which data is collected or generated is more widely accessible and thus collaborative. Yet, because the data, especially in health research, may relate to identifiable information about human subjects, researchers and other societal actors engaging in open science practices in Nigeria must do so in compliance with the existing legal frameworks on privacy and other related rights such as human dignity. Similarly, the sharing of biological samples in health research may require researchers and other societal actors adopting open science practices to pay attention to privacy rights. Thus, the discussion in the following section introduces the various pieces of legislation, regulations and guidelines governing privacy rights and data protection in Nigeria and analyses their provisions in relation to open science in health research. In doing this, the study also highlights the implications of privacy rights and data protection for researchers subscribing to open science in health research.

## Legal frameworks

In Nigeria, the privacy, dignity and other fundamental rights and freedoms of natural persons are guaranteed under Chapter IV of the Constitution of the Federal Republic of Nigeria, 1999 (as amended to 2023) [12]. The Nigeria Data Protection Act (NDPA) 2023 [13] builds on the constitutional provisions to ensure the privacy rights of persons and their other fundamental rights in relation to the processing of their personal data. In this respect, personal data is defined as ‘any information relating to an individual, who can be identified or is identifiable, directly or indirectly, by reference to an identifier’.^3^ An identifier could be a name, an identification number, location data, an online identifier or one or more factors specific to the physical, physiological, genetic, psychological, cultural, social, or economic identity of a person [13].

When carrying out scientific research in relation to personal data, researchers and other societal actors are to comply with the various principles governing data processing stipulated in the NDPA 2023, particularly where they are either domiciled, resident or operating in Nigeria, or the research involving the personal data occurs within Nigeria, or the human subject is in Nigeria [13]. This is because ‘processing’, as used under the Act, is a broad term covering the wide range of activities researchers carry out in relation to human data, including their generation, utilisation, adaptation, alteration, storage and transfer for research purposes, whether by automated means or not. A contravention of any data privacy principles, and provisions of the NDPA 2023 in general would lead to sanctions such as a fine or even imprisonment [13]. But, researchers may, to some extent, be exempted from the application of the NDPA 2023, where personal data processing is carried out for personal or household purposes, or for preventing or controlling a national public health emergency, like the Covid-19 pandemic, among others [13].

By virtue of section 64(2)(f) of the NDPA 2023, the implementation of the Act may be supported by the Nigeria Data Protection Regulation (NDPR) 2019 [14],^4^ Nigeria Data Protection Regulation 2019: Implementation Framework (Implementation Framework 2020) [15] and other regulatory frameworks [16]. Apart from these, there are additional legal frameworks on privacy and data protection specifically relating to health research and practices. In this regard, the National Health Act 2014 (NHA 2014) [17], among other things, sets out the rules and standards for the rendering of health services in Nigeria and related matters such as health research, including those on confidentiality, access to and protection of health records. Specifically, section 33 of the NHA 2014 establishes the National Health Research Ethics Committee (NHREC), which periodically issues guidelines in furtherance of the privacy and other legal rights of human subjects in the context of health research. These include the National Code of Health Research Ethics 2007 (NCHRE 2007) [18], which, beyond data privacy, promotes the protection of human privacy in terms of biological samples, and the Policy Statement on Storage of Human Samples in Biobanks and Biorepositories in Nigeria 2013 (Policy Statement 2013) [19], which focuses on the storage and sharing of human biological samples.

From the international perspective, it is worth noting that as a member of the African Union (AU) and Economic Community of West African States (ECOWAS), Nigeria may also be influenced by their data protection instruments – AU Data Policy Framework 2022 [20], AU Convention on Cyber Security and Personal Data Protection 2014 (Malabo Convention) [21] and the ECOWAS Supplementary Act on Personal Data Protection 2010 [22]. While there may have been no direct legislative response to the instruments in line with section 12(1) of the Constitution 1999 (as amended to 2023) [12], their imports can be said to have been addressed by the existing legal regimes on data privacy in Nigeria.^5^ Significantly, section 5(i) of the NDPA 2023 provides that the National Data Protection Commission shall ensure compliance with Nigeria’s international personal data protection obligations and best practices [13]. Similarly, paragraph 4.1.1 of the Implementation Framework 2020 [15] also provides that the Malabo Convention 2014, as well as the European Union General Data Protection Regulation 2016 (EU GDPR) [24], may be of persuasive value in Nigeria, but only to the extent that there are no existing provisions for a data protection principle or process.

To understand the implications of the forgoing legal instruments on privacy and data protection for researchers seeking to adopt an open science approach to health research in Nigeria, the following discussion highlights some privacy and data principles relating to open science in health research.

### Consent is required

Based on the existing regulatory frameworks on privacy and data protection in Nigeria, researchers are generally required to, among other things, obtain the informed consent of human subjects before processing their personal data or samples.^6^ Under the NDPA 2023, the researcher bears the burden of proof for establishing that the consent of a human subject had been sought for and obtained.^7^ But it is worth mentioning that consent may not be required to process data in circumstances where it is necessary:

- for the performance of a contract to which the data subject is a party or to take steps at the request of the data subject prior to entering into a contract,
- for compliance with a legal obligation to which the data controller or data processor is subject,
- to protect the vital interest of the data subject or another person,
- for the performance of a task carried out in the public interest or in the exercise of official authority vested in the data controller or data processor, or
- for the purposes of the legitimate interests pursued by the data controller or data processor, or by a third party to whom the data is disclosed.^8^

Significantly, human subjects have the right to object to the processing of their data,^9^ or even withdraw their consent at any given time.^10^ However, when exercised, consent withdrawal does not affect the lawfulness of that which has already been processed.^11^ While the Policy Statement 2013 supports broad consent with regard to the ‘receiving, handling, storage and distribution’ of biological samples in biobanks,^12^ the NDPA 2023 provides for specific consent, in that the consent as obtained must not only be freely given, it must be ‘specific’ and ‘unambiguous’.^13^ In other words, it must be for specific purpose or purposes. Because consent is obtainable only for specific purposes, the extent to which researchers can process personal data further or reuse the data collected for secondary research remains an open question that this study attempts to address in subsequent discussions.

It is important to note that, by virtue of section 26 of the NDPA 2023, a researcher cannot consider the silence or inactivity of a human subject as constituting consent, whether for the initial or further data processing. Consent must be affirmative, either in writing, orally or electronically [13].

### Further Requirements

In addition to the above rules regarding consent, researchers must also adhere to the general principles of personal data processing contained in section 24 of the NDPA 2023. Among other things, researchers must ensure that personal data are:

- processed in a fair, lawful and transparent manner;
- collected for specified, explicit, and legitimate purposes, and not to be further processed in a way incompatible with these purposes;
- adequate, relevant, and limited to the minimum necessary for the purposes for which the personal data was collected or further processed;
- retained for not longer than is necessary to achieve the lawful bases for which the personal data was collected or further processed;
- accurate, complete, not misleading, and, where necessary, kept up to date having regard to the purposes for which the personal data is collected or is further processed; and
- processed in a manner that ensures appropriate security of personal data, including protection against unauthorised or unlawful processing, access, loss, destruction, damage, or any form of data breach [13].

Prior to collecting personal data ‘directly’ from a human subject, a researcher is required to inform the human subject about ‘the purposes of the processing for which the personal data are intended’, ‘recipients or categories of recipients of the personal data, if any’, ‘retention period for the personal data’, and the ‘existence of automated decision-making’ such as profiling’, among others.^14^ Similarly, before initiating the further processing of data, a researcher must, in the same manner, provide the human subject with the stipulated information except where the human subject has already been provided with such information or provision of the information is ‘impossible or would involve a disproportionate effort or expense’.^15^

The NDPA 2023 [13] also sets out provisions on cross-border data flow^16^ and rights of data subjects^17^, and the right to data portability,^18^ among others. Similarly, the NCHRE 2007 [17] sets out an elaborate provision regarding the international transfer of samples, requiring details of the ‘type of materials, anticipated use, location of storage outside Nigeria, duration of such storage, limitations on use, transfer and termination of use of such materials subject to any law, regulations and enactment in Nigeria’.^19^

## Implications of privacy and data protection rules on the adoption of open science by health researchers in Nigeria

All of the foregoing privacy and data protection rules raise the question of whether privacy rights are incompatible with the open science process – especially in the context of health research. What are the implications of the application of privacy rules to open science? Do the regulations on privacy not create legal barriers to the collection, analysis, utilisation and sharing of data, samples and other research outputs in open science? These questions are crucial for various reasons. For one, scientific research is generally dynamic, with the potential to take entirely different directions from that which could have been anticipated [25].^20^ This means that the chances that researchers may need to use data or samples collected in other contexts are high, but this may not be known at the time of procuring consent. More specifically, as data and samples under the open science framework are openly accessible, there are also chances that recipients will use them for purposes that are not necessarily connected or compatible with the initial study for which the researchers or research institutions processed the data. While all these circumstances conflict with the purpose limitation principle, there is no gainsaying that requiring researchers to procure consent for diverse and subsequent purposes in open science places an unbearable burden on them.

Also, in light of the above, researchers seeking to adopt an open science approach to health research may not be able to meet the obligation of providing all the information required under section 27 of NDPA 2023 [13].^21^ Aside from being unable to provide information about the future use of the data, researchers may also not be able to fulfil the requirement of providing information about potential users or recipient(s) or categories of recipients of the personal data. This is of critical importance in open science, where it may be impossible to identify potential or actual recipients due to the promotion of universal access to data. In this context, the recipients may also gain access to datasets more than is necessary for their own research purposes. Furthermore, the inclusiveness of the open science model also engenders dynamic and unending research cycles under which the availability and use of data, as well as samples, may be for a period longer than envisaged [26, 27]. Yet researchers, in procuring consent, need to provide information regarding the duration of research or storage of data/samples. All of these issues raise the concern of whether consent if obtained in the context of open science, could be said to be *informed*.

Quite significantly, section 24(1)(b) the NDPA 2023 limits the further processing of data to purposes that are compatible with the initial purpose for which the data was processed. Sub-section 4 provides for compatibility assessment, while it is also stipulated that further processing for scientific purposes, ‘shall not be considered to be incompatible with the initial purpose’. Even then, there is no clarity on whether the data processing principles, such as purpose limitation, data minimization and storage limitation, apply to the secondary processing of data for scientific purposes. Also, it is not clear whether researchers in further processing data for scientific purposes could do so without obtaining consent or establishing any of the other lawful basis, the data having initially been collected based on consent or through any lawful means. Although, it may be safe to argue that, as consent is obtained for specific purpose or purposes, a further processing beyond that which is covered under the initial consent would require a separate lawful basis, it is challenging in the open science context where rapid and unrestricted data access is promoted. More so, section 27(2) of the NDPA 2023 requires the provision of information to data subjects as regards the processing of data not directly collected from them in the same manner as when the data is collected directly, with the possible exceptions set out. The human subject may consent to the further processing of the data based on the information provided.^22^

It is worth noting that, Article 2.1(1)(a)(i) of the NDPR 2019 and paragraph 4.1.2 (b-c) of the Implementation Framework 2020 suggest that researchers could legitimately engage in further processing of personal data without obtaining separate consent from the human subject if it is solely for scientific or historical research, statistical purposes which are for public interest or such further processing of the data is required in compliance with an existing legal obligation. The extent to which these provisions allow researchers to share and use health data in the public domain freely or if it accommodates the various open practices highlighted in the discussions so far is highly doubtful. This is because it is further stipulated that a person or entity processing data for scientific research purposes ‘shall not transfer any personal data to any person’.^23^ By this, the adoption of open science in health research in Nigeria is truncated, given the centrality of data sharing. Also, while the NDPA 2023, as well as the NDPR 2019, emphasize compatibility in the further processing of data, with the secondary use of data for scientific purposes permissible, it is worth stating that data is not necessarily made open to the public within the open science context only for scientific research purposes – but for ‘any responsible purpose’ [28], ‘scientific or not’ [29].

Underlying privacy rights and the principle of informed consent is the ability of human subjects to, among other things, exercise their right to object to the processing of their personal data, withdraw consent, and request that their data be erased or that their samples are withdrawn from research. But it has been observed, in the aspect of data privacy, that because ‘data are distributed via open access it is impossible to ensure that the data are deleted or removed at a later time, which would mean that the withdrawal of consent is practically ineffective’ [29]. Wiebe and Dietrich [29] further pointed out that:

to effectively comply with the obligations created by a potential withdrawal, they need to implement measures to make any future withdrawal technically possible. But in an open access environment such as an open repository, it is not desirable, if not impossible, to implement technical measures to ensure that data are deleted or removed after a withdrawal.

This may affect the level of trust that human subjects may repose in the informed consent process and their willingness to permit the processing/further processing of their personal data within the open science context generally.

All the foregoing legal issues suggest that the existing regulatory frameworks on privacy rights and data protection could impede the adoption and sustainability of open practices in health research in Nigeria to the extent that they restrict the sharing and use of identifiable data and samples. As current health realities demand open collaborative research, possible legal options through which an open approach to health research could be fostered among researchers and research institutions in Nigeria are explored later in this study.

### The International Dimension

Like the NDPA 2023, it suffices to note that the EU GDPR 2016 [24] similarly provides for the further processing of data for scientific research purposes, and this is understood as creating an exception to the purpose limitation and storage limitation rules.^24^ But the scientific research exemption under the EU GDPR 2016 is not without its limits as it is expressly subject to ‘appropriate safeguards’, which among other things, are to ensure respect for data minimization [24]. Also, the exemption is not applicable where the researcher could have fulfilled the purpose for which personal data is further processed through the use of anonymous data [24]. In other words, it is not in all cases that the EU GDPR 2016 permits the secondary use of personal data for research purposes. Furthermore, in some jurisdictions, the exceptions with regard to the principles of purpose and storage limitations may apply exclusively to scientific research institutions, in which case, when any other entity further processes data for research purposes, it must do so in compliance with all the data privacy principles [30]. But again, open science actors may use open data for purposes other than scientific research. Also, as suggested, the requirement to put in place ‘appropriate safeguards’ to ensure the restrictive data protection principles would seem to be at odds with the principle of openness [29].

The privacy rules on the international transfer of personal data or samples are also critical, as open science emphasizes scientific collaboration and the sharing of resources across national boundaries. Particularly, they have significant implications for Africa, where collaborative research to address diseases affecting the continent is being promoted. The NDPA 2023 [13] and NCHRE 2007 [18] make it legally possible for researchers to transfer data and samples to researchers in foreign countries or international organisations, subject to the existing rules on privacy.^25^ Concerning the transfer of personal data, it is specifically stipulated that the recipient must be subject either to a law, binding corporate rules, contractual clauses, code of conduct, or certification mechanism that affords an adequate level of data protection.^26^ Some of the criteria for assessing the adequacy of the nature of data protection are specified in section 42 of the NDPA 2023, and these include the ability of human subjects to enforce their rights through administrative or judicial redress.^27^ In addition to the adequacy rule, there are other legal bases through which data may be transferred outside Nigeria to enable data access and foster collaborative research in the continent and globally. These are contained in section 43 of the NDPA 2023. For example, the human subject may consent to the proposed transfer after being informed of the possible risks of such transfers or the transfer is considered necessary for important reasons of public interest. However, as with other legal provisions on privacy and data provision, recipient countries/researchers may find the adequacy requirement and other requirements for international data transfer restrictive.

Apart from the transfer of data and samples from Nigeria to other countries, as discussed above, it is also essential in open science research that health researchers in Nigeria are able to access data and samples from other countries. With specific regard to data protection, various countries, such as those of the EU, also have legal requirements for cross-border data transfer, with third countries similarly required to have an adequate level of data protection [24]. Exceptions such as the consent of the human subject and fulfilment of contracts are also provided for [24]. However, Nigeria is yet to be recognized by the EU as providing the appropriate level of protection for data [31]. The effect is that the transfer of data to researchers in Nigeria is restricted from researchers in the EU and other countries with a similar point of view that Nigeria does not guarantee an adequate level of protection. This could impede the advancement of open science in health research in a developing country such as Nigeria. More specifically, the possibility of researchers in Nigeria engaging in open collaborative health research with their peers in Africa and other jurisdictions is also restricted.

## Options for the Adoption of Open Science in Health Research in Nigeria

From the foregoing, it appears that open science in the context of health research may not be easy to institutionalize among Nigerian researchers and their open science collaborators within and outside Nigeria as the existing legal principles on privacy rights and data protection tend to restrict the collection, access and use, as well as dissemination of the data and samples of human subjects. Nevertheless, to mitigate possible infringements by researchers and the impact of privacy and data protection rules on open science, this study identifies four possible legal options for advancing open health research among researchers in Nigeria and their international collaborators.

### a. Use of Anonymous Data/Samples and Synthetic Data:

The Nigerian privacy and data protection rules do not apply to health research activities involving data and samples that are not identifiable in nature or from which human subjects are no longer identifiable^28^ Thus, as a measure, researchers or institutions aiming to promote open science in health research would need to ensure that all personal identifiers that could allow the re-identification of human subjects by recipients or other researchers are removed prior to the sharing of health data for further processing or research. While this could foster open data/science models for health research and innovation without undermining the privacy and other related rights of human subjects guaranteed under existing legal regimes in Nigeria, it depends on the extent to which relevant health data can be anonymized. Also, it is worth noting that the anonymization of data remains a highly contentious issue with regard to scientific research, given the possibilities of reidentification. With specific regard to open science research, it is believed that because of the open sharing of data, ‘the likelihood that any other person will have the means and will use those means to re-identify the data subjects increases very significantly’ [29].

In addition to anonymous data, the use of synthetic data^29^ for health research, and other purposes, is increasingly being considered a mechanism for addressing data privacy concerns [31, 32].^30^ This is because it is believed that privacy risks tend to be minimal in the use and sharing of *fully synthesised* data as it ‘contains no identifiable information about the dataset it was generated from and is considered a safe approach for the sharing of sensitive data’ [32]. In this regard, open science practices are feasible with the privacy and data protection rules not applicable because synthetic data would not be considered identifiable personal data [31]. Notwithstanding, because of the possibility of privacy breaches regarding synthetic data generated from personal data, *privacy assurance assessments* are encouraged to evaluate the extent to which real people may be matched to synthetic data [31].

### b. Health Research for Public Interest:

Notably, section 45 of the Nigerian Constitution 1999 (as amended to 2023) [12] permits the derogation of privacy rights by the government in public interests, including health. In other words, data and samples collected and processed in furtherance of public interest or public health may be exempted from privacy and data protection.^31^ Given that open science is public interest-driven and considered ‘a global public good’,^32^ there are chances that the public interest exception to privacy rights and data protection can be used to promote open science in health research and allow researchers to freely process and disseminate identifiable and non-identifiable data/biological material without obtaining the consent of human subjects. But, more significantly, the NDPA 2023 and other related legal instruments could be amended, explicitly recognizing sharing of data and biological materials for open science purposes as a matter of public interest. For now, it is essential that research institutions and researchers, particularly those that are publicly funded, ensure that their open science policy and practices are in furtherance of public health in Nigeria and not-for-profit. Even then, it is worth noting that the exercise of constitutional or statutory powers excluding public health research could still raise concerns about the encroachment on the privacy rights of human subjects by the government or its agencies, given the sensitive nature of health data.

### c. Personal Data/Material Transfer Arrangements (DTA/MTA):

Internationally, countries opt for special personal data or material transfer arrangements with countries or international organizations that they consider do not meet their adequacy requirements [34]. Such arrangements may, among other things, require a recipient to commit not to re-identify human subjects.^33^ To gain access to anonymous data and biological samples from other countries in order to promote open science within the health research context, it is suggested that research institutions in Nigeria explore similar arrangements, subject to putting in place relevant safeguards to engender trust and confidence from their international research collaborators.^34^

In addition, as open science is considered a global public good, international organizations could play an active role in supporting and promoting principles that portray or recognize the international transfer of data and biological materials for open science purposes as a matter of public interest within the frameworks of privacy and data protection. This could influence the various national privacy and data protection rules on cross-border transfer and facilitate international cooperation in health research.

### d. Privacy Rights Waiver by Human Subjects:

The increasing practices of open science which rely on the free flow of health data, are raising the critical question of whether human subjects could choose to relinquish their data privacy right or waive the principle of informed consent to facilitate an open approach to health research. In other words, as privacy rights confer on human subjects the power to control (and even object to) the processing of their data, should they not have the freedom to decide whether to donate their personal data and waive the need for consent to allow unrestricted access, use and sharing, particularly for open health research purposes? This envisages a possibility of human subjects voluntarily disseminating or permitting the processing and dissemination of their personal data in the public domain or on open science platforms, such that researchers could freely access it for health research without the need to seek and obtain consent – provided the research is deemed appropriate by the relevant Health Research Ethics Committee.

Currently, the rules on privacy and data protection in Nigeria, as discussed in the preceding sections, appear to foreclose interpretations that could confer on human subjects the power to waive the need for researchers to seek consent, even regarding personal information in the public domain. This is because they are quite explicit regarding the need for researchers to seek and obtain the consent of human subjects and other legal bases for processing and further processing personal data. Thus, this study calls for the adoption of a set of data protection rules that expressly expand the exceptions to consent requirements to include the processing of data that is placed in the public domain by human subjects or that human subjects have allowed to be disseminated in the public domain by researchers and data controllers in general.^35^

The foregoing could minimize the privacy and data protection constraints and provide greater leeway for open science practices. In this regard, promoting an understanding of open science benefits among human subjects is necessary. While the need to incentivize open science practices among researchers is well acknowledged, although not necessarily addressed from a practical perspective,^36^ there is also a need to explore mechanisms that could incentivize human subjects and their communities to accept open practices in relation to their personal data.

## Conclusion

The trend toward open science emerging in Nigeria and other countries around the world is raising critical privacy and data protection issues for research institutions and researchers engaging in health research. As shown, the requirements of *informed* consent and other underlying principles of privacy rights and data protection designed to protect human subjects render the open sharing of genetic or health-related data practically impossible in the context of open science research. Notwithstanding this constraint, researchers who subscribe to the principles of open science can still explore other legal options to reduce the risks of violating the existing privacy and data protection rules. Significantly, this study suggests the adopting of privacy and data protection rules that recognize data sharing in the context of open science as a public interest and confer human subjects with the power to waive consent, particularly regarding personal information that they have voluntarily allowed to be processed and disseminated in the public domain/open science platforms. Until such rules are developed, researchers can, in the meantime, rely on data anonymization, DTAs/MTAs and the legal provisions on public interests as the legal basis for processing personal data for health research in the open science context. As open science continues to evolve among researchers in Nigeria and around the world, there is a continuing need to seek ways to reconcile the underlying goals of privacy and data protection with those of open science.

## Data Availability

Not applicable.

## Footnotes

1. See paragraph II.12 UNESCO Recommendation on Open Science 2021.

2. See paragraph 3.13 NSTIP 2022.

3. See section 65 of the NDPA 2023.

4. Prior to the enactment of the NDPA 2023, the Nigeria Data Protection Regulation 2019, was generally recognized as the principal regulatory framework on data privacy in Nigeria. See Incorporated Trustees of Digital Rights of Lawyers Initiative v. FRN, ECW/CCJ/JUD/02/23 (2023) & Incorporated Trustees of Laws and Rights Awareness Initiative and Ors v NIMC, LPELR-55623 (2021) (CA).

5. See Incorporated Trustees of Digital Rights of Lawyers Initiative v. FRN (2023) ECW/CCJ/JUD/02/23.

6. See sections 25(1)(a), 26 and 30(1)(a) of the NDPA 2023; sections 28(1)(b), 32, 48 and 55–56 NHA 2014; Part 2 of the NDPR 2019; Section F(f) of the NCHRE 2007; Policy Statement 2013.

7. See section 26(1) of the NDPA 2023.

8. See section 25(1)(b) of the NDPA 2023; See also section 30(1)(b-i) of the NDPA 2023 and Article 2.2 (b-e) of the NDPR 2019.

9. See section 36 of the NDPA 2023; Article 2.8 of the NDPR 2019.

10. See sections 26(4) and 35 of the NDPA 2023.

11. Section 26(5) of the NDPA 2023; Articles 2.3(2)(c) and 3.1(7)(i) of the NDPR 2019; Sections E(n)(6) and F(g) of the NCHRE 2007.

12. 12. Section E.1 Policy Statement 2013.

13. Sections 25(1(a) and 65 of the NDPA 2023; Articles 1.3(iii) and 2.1(a) of the NDPR 2019.

14. See section 27(1) of the NDPA 2023.

15. See section 27(2) of the NDPA 2023.

16. See sections 41–43 of the NDPA 2023; Article 2.11 of the NDPR 2019.

17. See section 34 of the NDPA 2023; Article 3.1 of the NDPR 2019.

18. See section 38 of the NDPA 2023.

19. See section E(n) NCHRE 2007.

20. See Recital 33 of the EU GDPR acknowledging that: ‘It is often not possible to fully identify the purpose of data processing for scientific research purposes at the time of data collection ‘.

21. See also paragraph 7 of Article 3.1 of the NDPR 2019; Section F(5) of the NCHRE 2007, also requires that an informed consent document should contain information about the purpose(s) of research.

22. See paragraph 4 1.2(a) of the Implementation Framework 2020.

23. See Article 2.1(1)(a)(ii) of the NDPR 2019.

24. See recital 50, Article 5(1)(b) and (e) and Article 89(1) of the EU GDPR 2016.

25. See section 41 of the NDPA 2023; Article 2.11 of the NDPR 2019; section E(n) of the NCHRE 2007.

26. See article 2.11(a) of the NDPR 2019.

27. A list of countries believed to guarantee an adequate level of data protection is contained in Annexure C of the Implementation Framework 2020. It includes all African countries and country members of the EU implementing the Malabo Convention 2014 and EU GDPR 2016, respectively.

28. See section 28(2) NHA 2014; paragraph 2.1(iii) of the Implementation Framework 2020.

29. According to Khaled, Mosquera, and Hoptroff, synthetic data ‘is not real data, but data that has been generated from real data and that has the same statistical properties as the real data’. Thus, the results generated from synthetic data should be similar to those generated from the real data. For further conceptual clarification, see, El Emam K, Mosquera L, Hoptroff R. Practical synthetic data generation: balancing privacy and the broad availability of data. O’Reilly Media; 2020 May 19.

30. 30. Dankar and Ibrahim pointed out that at a 2020 future of privacy forum, synthetic data generation was cited as ‘a crucial privacy enhancing technology for the coming decade’. Dankar FK, Ibrahim M. Fake it till you make it: Guidelines for effective synthetic data generation. Applied Sciences. 2021 Feb 28;11(5):2158.

31. See also Section 25(1)(b)(iv) of the NDPA 2023; Article 2.2(e) of the NDPR 2019; paragraph 2.1(i) of the Implementation Framework 2020.

32. See paragraph III.13(b) of the UNESCO Recommendation on Open Science 2021.

33. Ibid.

34. For a model DTA/MTA, see: Swales L, Ogendi P, Botes M, Townsend B, Donnelly D, Abdulrauf L, Thaldar D, A Data Transfer Agreement Template for South Africa (1.0) (2022). https://doi.org/10.5281/zenodo.7537396. Accessed 20 Jun 2023; Mendy M, Caboux E, Lawlor RT, Wright J, Wild CP. Common Minimum Technical Standards and Protocols for Biobanks Dedicated to Cancer Research. (IARC Technical Report, No. 44.) ANNEX 4. Template Material/Data Transfer Agreement (MTA/DTA) (2017). https://www.ncbi.nlm.nih.gov/books/NBK567253/. Accessed 20 Jun 2023.

35. In Zimbabwe, Section 12(3)(g) of the Data Protection Act, 2021, permits the processing of genetic, biometric and health data without consent where the data subject has made them public; see, also, paragraph 30(5) of the Tanzanian Protection of Personal Information Bill of 2022.

36. Paragraph IV.17 of the UNESCO Recommendation on Open Science 2021.

## Abbreviations

AU: African Union
Covid-19: Coronavirus Disease
ECOWAS: Economic Community of West African States
EU: European Union
GDPR: General Data Protection Regulation
NCHRE: National Code of Health Research Ethics
NDPA: Nigeria Data Protection Act
NDPR: Nigeria Data Protection Regulation
NHA: National Health Act
NSTIP: National Science, Technology and Innovation Policy
UNESCO: United Nations Educational, Scientific and Cultural Organization
